# Family-based weight stigma and psychological well-being of adolescents: a longitudinal analysis of recent vs. cumulative exposure

**DOI:** 10.3389/fpsyt.2025.1623411

**Published:** 2025-10-03

**Authors:** Dimitra Anastasiadou, Salomé Tárrega, Albert Fornieles-Deu, David Sánchez-Carracedo

**Affiliations:** ^1^ Department of Clinical and Health Psychology, Universitat Autònoma de Barcelona, Bellaterra (Cerdanyola del Vallés), Barcelona, Spain; ^2^ Department of Epidemiology and Methodology of Social and Health Sciences, Faculty of Health Sciences at Manresa, Universitat de Vic - Universitat Central de Catalunya (UVic- UCC), Manresa, Barcelona, Spain; ^3^ Department of Psychobiology and Methodology of Health Sciences, Universitat Autònoma de Barcelona, Bellaterra (Cerdanyola del Vallés), Barcelona, Spain

**Keywords:** weight stigma, families, adolescents, well-being, longitudinal

## Abstract

**Introduction:**

Family-based weight stigma has been linked to adverse psychological outcomes in adolescents. Research on weight stigma in the Mediterranean area is scarce. This study aims to longitudinally explore the association between family-based weight stigma and adolescents’ psychological well-being, considering recent vs cumulative exposure.

**Methods:**

Data from the two-year longitudinal WbSad study were drawn from baseline assessments (T1) of a representative sample of 1,016 secondary school adolescents in a large Spanish city. At follow-up (T2), 551 adolescents participated. The mean age at T2 was 15.8 years, with 48.5% girls. Multivariate linear regression models, adjusting for relevant covariates and baseline values, examined the impact of exposure (Never, Only at T1, at T1 and T2, or Only at T2) to family-based weight stigma and to parental comments about weight and dieting on psychological outcomes, measured with the Depression Anxiety Stress Scale-21 (DASS-21) and the Rosenberg Self-Esteem Scale.

**Results:**

Family-based weight stigma was reported more frequently among girls and was associated with higher psychological distress. Girls exposed to family stigma (at T1 and T2, and Only at T2) reported higher psychological distress, with significant associations across all DASS-21 outcomes for those exposed at T2 only. Maternal comments were linked to greater distress and lower self-esteem in girls and higher stress and total distress in boys at T2 only. Paternal comments at T2 were significantly associated with higher depression and total DASS-21 scores in girls, and higher scores in all DASS-21 outcomes in boys. No significant associations were found between parental encouragement to diet and any psychological outcomes in either gender.

**Discussion:**

This study provides novel insights into how the timing (recency vs. persistent exposure) and source (maternal vs. paternal) of family-based stigma shape adolescent outcomes in a non-Anglo-Saxon sample. Recent family-based weight stigma negatively impacts adolescent psychological well-being, with girls being particularly vulnerable. The absence of an effect from cumulative exposure warrants further exploration. Preventive strategies should educate parents to avoid stigmatizing comments and promote messages that prioritize well-being over weight, particularly before the onset of mid-to-late adolescence. Finally, research is needed to better understand the temporal dynamics of parental weight-related comments and their impact on adolescents.

## Introduction

Individuals with higher weight experience pervasive stigma driven by cultural reinforcement of the thin ideal, negative social perceptions of them, and the blame-and-shame framing in media and public health, where their weight is attributed to personal responsibility (1). This stigma remains highly tolerated throughout the lifespan and across multiple domains of everyday life (2, 3). In youth, weight-based victimization —including teasing, bullying, and harassment— is highly prevalent and disproportionately affects children with higher body weight (4). In fact, body weight is reported as the most common reason for peer-based teasing and bullying, surpassing other forms of discrimination such as race/ethnicity, sexual orientation, or disability status (5).

Extensive evidence documents the negative impacts of weight stigma on youth’s physical, psychological, and social health (6–8). Psychological consequences include depression, anxiety, poor body image, disordered eating, substance abuse, and self-harming behaviors (9). Longitudinal research further highlights enduring negative health outcomes, showing that weight stigma contributes to weight gain over time regardless of initial weight status, race, or sociodemographic factors (10–12).

Weight stigma is exacerbated when stigmatized individuals internalize these negative attitudes, a phenomenon known as weight bias internalization (WBI). WBI is associated with decreased overall functioning and lower quality of life (13), and has also been documented among children and adolescents (14). Research among Spanish adolescents indicates that WBI is higher among girls compared to boys and more prevalent in adolescents with a higher zBMI-for-age (15).

Families play a critical role in adolescents’ self-esteem, body image, and lifelong health habits (16). However, familial dynamics can also foster weight stigma, posing significant risks to adolescents’ health and well-being (17, 18). Recent research has identified family members as the most common interpersonal source of weight stigma experienced by adolescents (19). In particular, weight-related conversations between parents and their children, including parental critical comments promoting the need for weight loss and dieting, are associated with negative psychological outcomes among children, such as anxiety, depressive symptoms, body dissatisfaction, unhealthy weight control behaviors, and WBI (17, 20–23). From adolescents’ perspective, 66% reported experiencing weight-based teasing or bullying from their parents, with higher prevalence among girls and those with BMI ≥95th percentile or <5th percentile compared to other weight categories (24, 25). Similarly, from parents´ perspectives, 93% of parents of higher-weight adolescents endorse moderate explicit weight bias (26).

Longitudinal evidence from Project EAT suggests that parental weight talk tends to persist over time with negative outcomes that may extend beyond adolescence into adulthood, especially in relation to disordered eating behaviors. Retrospective findings further indicate that exposure to family weight talk as a child is associated with enduring negative outcomes in adulthood, including lower self-esteem and body satisfaction, and heightened depressive and anxious symptoms (18, 27, 28). Additionally, cumulative encouragement to diet from parents has been significantly associated with adverse weight-related and psychosocial outcomes in young adults, such as unhealthy weight control behaviors, low self-esteem, body dissatisfaction and depression in females, and weight control behaviors, low self-esteem, and body dissatisfaction in males (29).

Extensive research on the cumulative burden of adversity (30) —including cumulative exposures to weight stigma in family contexts (29)— demonstrates clear dose–response links with health across the life span, including exposures beyond the family, such as peer victimization, community violence, and racism. In adolescence, adverse childhood experiences (ACEs) are common and strongly related to the first onset of psychiatric disorders (31), and a meta-analysis indicates that multiple ACEs in youth are associated with higher odds of adult obesity (32). Within this cumulative-risk perspective, sensitization theory posits that recurrent stigma progressively heightens reactivity via stress-sensitization and allostatic processes, such that each additional episode evokes stronger affective and cognitive responses, consistent with the dose–response evidence above (33). By contrast, desensitization/habituation theory (34) proposes that repeated comments can lose novelty and emotional salience —especially when normalized within family routines— so a recent episode may exert stronger impact than a long history of lower-intensity exposure. These considerations motivate our objective to compare the associations of recent versus cumulative family-based weight stigma with adolescents’ psychological outcomes. Moreover, stigma can be understood as a dynamic process unfolding across historical/structural context, human developmental stage, and status course, and underscores the understudied developmental timescale in shaping stigma experiences and health outcomes (35). Consistent with this view, developmental science identifies early–mid adolescence as a particularly sensitive period for stigma effects given heightened sensitivity to social evaluation. Classic theories describe the “imaginary audience”, a normative preoccupation with others’ judgments that is pronounced in early adolescence and decreases with maturation (36). Consistent behavioral and neurodevelopmental work indicates mid-adolescent peaks in social-evaluative reactivity, followed by a decrease in late adolescence as identity consolidates and regulatory control improves (37). In line with this work, studies on weight stigma report an age-graded decline in the prevalence of reported experiences from early/mid to late adolescence (38). However, to our knowledge, no study has directly compared adolescents’ social-evaluative reactivity to these experiences across early, mid, and late adolescence. Moreover, some cohorts show mid- to late-adolescent peaks in weight-stigma exposure and in negative self-judgments relative to early adolescence (15, 39).

Additionally, gender differences have emerged in the experience and impact of parental weight stigma. Maternal critical comments have been found to provoke stronger WBI in adolescents compared to paternal comments (40), though this gender difference has not been confirmed by Lessard and colleagues (17), who indicate that weight stigma from both parents is associated with poorer psychological health indicators in adolescents. Additionally, more girls than boys report weight teasing from family members (11), and parental weight talk is more strongly linked to disordered eating behaviors in girls than in boys (22).

The present study is part of the longitudinal WbSad project, which aims to describe the prevalence of weight stigma experiences and their internalization, and to explore their association with sociodemographic and psychological variables across two time points (T1 in 2022 and T2 in 2024) among a representative sample of secondary school adolescents from Spain. Initial findings from the WbSad study have been previously published (15).

Considering all the aforementioned, the main aim of the present study is to longitudinally explore how the frequency and recency of family-based weight stigma experiences (Never, Only at T1, at T1 and T2, and Only at T2) is associated with the psychological well-being of adolescents at T2. A secondary aim is to differentiate between weight stigma expressed by mothers versus fathers, examining their distinct impacts on adolescents’ well-being, as well as potential gender differences in adolescents´ responses.

## Materials and methods

### Design and participants

This is a two-year longitudinal survey-based study based on data from the WbSad study, a funded project on weight stigma in adolescents. As one of the main goals of the study was to obtain, for the first time in Spain, prevalence data of experienced and internalized weight stigma and its association with sociodemographic variables, in 2022 (T1), a representative sample of 1,016 adolescents (12–16 years) from the four courses of mandatory secondary education Spanish system was selected using random multistage cluster sampling (sampling error of 2.97% under the assumption of maximum indeterminacy and a confidence level of 95.5%; p = q = 0.5, 2σ). They came from 7 public and 9 grant-aided schools and one classroom for each course, with a total of 64 classrooms, coming from Terrassa, the third most populous city in Catalonia, Spain. Exclusion criteria were not having parental informed consent, not responding to the parental informed consent request, refusal to participate, or providing invalid answers because of language issues or failing the survey controls. Details of flow diagram of the sample at T1 can be found elsewhere (15). For data collection in 2024 (T2), a sample loss of approximately 50% was expected because participants who were in the last two years of the Spanish compulsory secondary education system at T1 would no longer be at that educational level two years later. They might have dropped out or continued studying other options such as high school or vocational training, which are usually attended at different institutions, making it difficult to locate these participants. Therefore, data collection at T2 focused on students who were in the first two years of secondary education at T1 (n = 519). Of these, a total of 422 students (81.3%) participated. Among these participants, 34 (6.6%) had dropped out of school and were not located in other participating schools, 29 (5.6%) did not attend class on the evaluation day, 23 (4.4%) did not pass the inventory controls, 8 (1.5%) did not give their informed consent, and 3 (0.58%) were excluded for other reasons. Despite the difficulties in doing so, a total of 129 students who were in the last two years of secondary education at T1 (n = 497) were located to participate in T2. Of these, most losses were due, as expected, to not being located in the participating schools because they had left the system (n = 351, 70.1%). Additionally, 16 (3.2%) were not present at the time of the evaluation, and 1 (0.2% did not pass the inventory controls. Finally, 551 (54.2%) adolescents from the same cohort of T1 participated again in T2, two years later.

### Procedure

The study was supported by the Community and Health Service of the City Council of Terrassa, which facilitated the sampling and contact with participating schools. Parental informed consent and participants’ assent were obtained at T1. The assessments in T1 were carried out in April and May 2022. The survey was administered over the course of one hour in the classrooms on an online platform of the company Digital Insights S.L. The assessment was supervised by a group of graduate psychologists previously trained. The survey employed forced responses and incorporated controls for response ranges and interspersed control questions to verify the level of attention of the participants, avoiding missing data. While the participants answered the survey, a group of 5–7 adolescents were moved to a private area, where anthropometric measurements were taken following a standardized protocol (41) and recommendations by the Catalan Public Health Agency to minimize any possible adverse effect (42). After that, the participants returned to the classroom and completed the survey. Data was pseudo-anonymized. Regarding the second measure, it took place in April and May 2024. The procedure was the same except that, since all participants were over 14 years old, they provided their informed consent, and it was not necessary to obtain parental informed consent, in accordance with article 7 of the Spanish Organic Law 3/2018 on the Protection of Personal Data and guarantee of digital rights. Nevertheless, the families were informed about the study. The WbSad study was conducted in accordance with the Declaration of Helsinki of the World Medical Assembly (43) and approved by the Ethics Committee of the author’s university for both the first (CEAAH 3451) and the second measure (CERec-6677). More details about the procedure can be found in (15).

### Instruments

#### Sociodemographics and anthropometrics

Participants provided information on their age, gender, parental origin, and socioeconomic status. Socioeconomic status was estimated using the Hollingshead Two-Factor Index of Socioeconomic Status (SES) (44), which combines the parents’ educational and occupational levels. Levels of SES were classified into low, medium-low, medium, medium-high, and high. Height (in cm) was measured using a SECA 214 portable stadiometer (20–207 cm; accuracy range of 0.1 cm) and weight (in kg) using a SECA portable scale (Model 8777021094) (0–200 kg; accuracy range of 0.1 kg). Weight status was then calculated based on z-BMI scores, in accordance with the World Health Organization growth reference criteria (45).

#### Experiences of family stigma

Assessments of Experiences of Family Stigma at T1 (2022) and T2 (2024) were based on a proposal of previous research to assess sources of stigma and their frequency (46). Participants were asked: “Have family members ever teased, harassed or treated you unkindly, or made you feel bad or uncomfortable because of your weight?” Response options ranged from 1 = Never; 2 = Rarely; 3 = Sometimes; 4 = Often to 5 = Very often. The internal consistency in our sample at T1 was α = 0.817 and ω = 0.817, and at T2 was α = 0.740 and ω = 0.736.

To construct exposure groups, responses were first recoded into a binary indicator at T1 and at T2: Never/Rarely as “No” and Sometimes/Often/Always as “Yes”. Based on these binary indicators at T1 and T2, we then created a four-category mutually exclusive exposure variable: Never (no exposure at T1 or T2), Only at T1 (earlier-only), at T1 and T2 (cumulative), and Only at T2 (recent).

#### Parental comments about weight and dieting

Adolescents’ perspectives on parental comments about weight and dieting were based on previous research focused on weight stigma in adolescents (40), which adapted a measure used in Project EAT (47). Adolescents were asked the following 3 questions: (1) “how often does your mother make comments to you about your weight?” and (2) “how often does your father make comments to you about your weight?”. Responses to these questions were rated on a 5-point scale from never to very often. Additionally, they were asked (3) “to what extent does your father or mother encourage you to start a diet to lose weight or avoid gaining weight?”, rated on a 5-point scale from not at all to very much. Responses were recoded into two categories: Never/Rarely or Not at all/Very little as “No” and Sometimes/Often/Always or Sometimes/Quite a lot/Very much as “Yes”. Using the same four-category scheme as above, we combined T1 (2022) and T2 (2024) to classify exposure as Never, Only at T1, at T1 and T2 (cumulative), or Only at T2 (recent).

#### Depression, anxiety, and stress scales (DASS-21)

The DASS-21 (48) is a 21-item self-report questionnaire designed to measure the severity of symptoms common to depression, anxiety, and stress. We used the Spanish validation (49). Participants rated on a Likert scale from 0 to 3 the intensity/frequency with which they experienced each of the 21 negative emotional symptoms that make up the questionnaire during the previous week. It contains 3 scales of 7 items each. The Depression scale evaluates sadness, lack of positive emotions, lack of enthusiasm and initiative to do things, self-devaluation, and lack of meaning in life (e.g., “I couldn’t seem to experience any positive feeling at all”). It has an internal consistency of 0.91. The Anxiety scale mainly evaluates somatic activation and worries about situations and the subjective experience of anxiety (e.g., “I felt scared without any good reason”). It has an internal consistency of 0.84. The Stress scale evaluates difficulty relaxing, hyperreactivity to situations, agitation, irritability, energy expenditure, and impatience (e.g., “I found it difficult to relax”). It has an internal consistency of 0.90. The final scores for each scale are multiplied by two, so the score range is from 0 to 42. Higher scores indicate more depression, anxiety, and stress. The internal consistency (α/ω) of the Depression, Anxiety, and Stress scales, and for the total score has been found to be 0.902/0.905, 0.857/0.859, 0.830/0.834, and 0.945/0.946 respectively for T1, and 0.892/0.858, 0.854/0.856, 0.835/0.839, and 0.941/0.941 for T2.

#### Rosenberg self-esteem scale

Self-esteem was assessed with the RSES (50) in its Spanish validation (51). It has 10 items (e.g. “I certainly feel useless at times”) that are answered on a scale from 1 (strongly disagree) to 4 (strongly agree). With a unidimensional structure, the internal consistency ranged from 0.85 to 0.88. The internal consistency in our sample at T1 was α = 0.890 and ω = 0.892, and at T2 was α = 0.885 and ω = 0.887.

#### Weight bias internalization

The Modified Weight Bias Internalization Scale (WBISM) (52) in its Spanish validation for adolescents (53) was used as an adjustment variable. It measures WBI across the body weight statuses (e.g., “I hate myself for my weight”). This version has 10 items with responses rated on a 7-point Likert scale (from strongly disagree to strongly agree). The mean of the item responses serves as the participant’s score (range 1–7), with higher scores indicating higher WBI. The Spanish validation of WBISM for adolescents of WBISM has showed a high internal consistency (α = 0.93; ω = 0.93) and showed a unidimensional structure with an adequate fit. The internal consistency in our sample at T1 was α = 0.941 and ω = 0.946, and at T2 was α = 0.944 and ω = 0.949.

### Statistical analysis

Statistical analyses were conducted using STATA version 18. The significance level was set at 0.05, and all hypothesis tests were two-tailed. Analyses were stratified by gender. Sociodemographic, anthropometric characteristics, outcome variables (including DASS-21 subscale scores and total score, and Rosenberg Self-Esteem Scale score) at T1 and T2, and predictors —Experiences of Family Stigma and Parental Comments about Weight and Dieting (coded as: Never, Only at T1, at T1 and T2, Only at T2)— were described using frequencies and percentages for categorical variables, and means and standard deviations (SD) for continuous variables. Gender differences were assessed using Pearson’s Chi-Squared test for categorical variables and linear regressions for continuous variables, as appropriate. Multivariate linear regression models were used to examine the associations between predictors and outcomes at T2. All models were adjusted for relevant covariates, including baseline scores (T1) of the corresponding outcome, age, BMI z-scores at T1, European origin, socioeconomic status, internalized weight stigma (WBISM) at T1. Adjusted estimated means, Regression coefficients (B) with their 95% confidence intervals, and the R^2^ value were used to express the proportion of variance explained by the model. Preliminary diagnostic tests indicated heteroskedasticity and non-normally distributed residuals (p <.001 for skewness and kurtosis tests). Therefore, all regression models were estimated using robust standard errors. For models involving Experiences of Family Stigma, analyses were conducted and reported only for the female subsample due to the limited number of boys who reported such experiences. However, for exploratory purposes, adjusted estimated means were also plotted for the male subsample to allow visual comparison with the models conducted in girls.

## Results

### Sample description

Descriptive statistics of sociodemographics and weight status, stratified by gender, were focused on the sample at T2 and are presented in **Table 1**. The mean age of participants was 15.8 years (SD = 1.04), and 48.5% were girls. No participants identified as non-binary. No statistically significant gender differences were observed in sociodemographic variables. However, girls showed significantly higher internalized weight bias scores (WBISM) than boys. Descriptive statistics for Experiences of Family Stigma and Parental Comments about Weight and Dieting, as well as for the outcome variables, are shown in **Table 2**. Gender differences were observed in exposure to both Experiences of Family Stigma and Maternal Comments about Weight, with girls reporting higher exposure overall, particularly at T2. Regarding the outcome variables at T1 and T2, girls showed worse scores across all measures. Specifically, they reported higher levels of psychological distress on all DASS-21 subscales (Depression, Anxiety, and Stress) and lower self-esteem scores on the RSES compared to boys.

**Table 1:** Sample description stratified by gender at T2 (n=551).

	Gender		Sig.
	Girls	Boys	
N	267 (48.46%)	284 (51.54%)	
Age (Years) *mean (SD)*	15.75 (1.05)	15.79 (1.04)	0.634
Parental origin (ethnicity)			
Europe	207 (77.5%)	214 (75.4%)	0.548
Other	60 (22.5%)	70 (24.6%)	
SES			
Low	13 (4.9%)	8 (2.8%)	0.209
Middle–low	44 (16.5%)	38 (13.4%)	
Middle	57 (21.3%)	77 (27.2%)	
Middle–high	92 (34.5%)	85 (30.0%)	
High	61 (22.8%)	75 (26.5%)	
WBISM *mean (SD)*	2.77 (1.62)	1.94 (1.25)	<0.001
Weight Status (WHO)			
zBMI < -2 SD	1 (0.4%)	10 (3.5%)	0.026
zBMI between -2DS and 1SD	216 (80.9%)	208 (73.2%)	
zBMI between 1DS and 2SD1	39 (14.6%)	50 (17.6%)	
zBMI > 2SD2	11 (4.1%)	16 (5.6%)	

T1, Time 1; T2, Time 2; SD, Standard deviation; Sig., Statistical significance. ^1^ Equivalent to BMI 25 kg/m^2^ at 19 years. ^2^ Equivalent to BMI 30 kg/m^2^ at 19 years.

**Table 2:** Descriptives of the predictors, and outcomes at T1 and T2, stratified by gender (n=551).

	Gender		Sig.
	Girls	Boys	
n	267 (48.46%)	284 (51.54%)	
Experiences of Family Stigma			
Never	190 (71.2%)	250 (88.0%)	<0.001
Only at T1	16 (6.0%)	9 (3.2%)	
at T1 and T2	26 (9.7%)	4 (1.4%)	
Only at T2	35 (13.1%)	21 (7.4%)	
Maternal Comments about Weight			
Never	170 (63.7%)	221 (77.8%)	<0.001
Only at T1	28 (10.5%)	28 (9.9%)	
at T1 and T2	32 (12.0%)	9 (3.2%)	
Only at T2	37 (13.9%)	26 (9.2%)	
Paternal Comments about Weight			
Never	202 (75.7%)	232 (81.7%)	0.062
Only at T1	24 (9.0%)	29 (10.2%)	
at T1 and T2	17 (6.4%)	8 (2.8%)	
Only at T2	24 (9.0%)	15 (5.3%)	
Parental Comments about Dieting			
Never	197 (73.8%)	205 (72.2%)	0.613
Only at T1	28 (10.5%)	34 (12.0%)	
at T1 and T2	16 (6.0%)	23 (8.1%)	
Only at T2	26 (9.7%)	22 (7.7%)	
DASS21 Depression, *mean (SD)*			
T1	14.40 (11.46)	7.21 (8.52)	<.001
T2	12.54 (10.39)	6.18 (7.30)	<.001
DASS21 Anxiety, *mean (SD)*			
T1	14.20 (10.93)	7.23 (7.10)	<.001
T2	12.76 (9.93)	5.96 (6.65)	<.001
DASS21 Stress, *mean (SD)*			
T1	15.27 (9.99)	9.59 (7.94)	<.001
T2	14.83 (9.34)	9.38 (8.26)	<.001
DASS21 Total, *mean (SD)*			
T1	43.87 (30.13)	24.03 (21.36)	<.001
T2	40.13 (27.24)	21.52 (19.68)	<.001
RSES, *mean (SD)*			
T1	27.66 (6.47)	32.50 (5.77)	<.001
T2	29.09 (6.62)	33.89 (4.85)	<.001

T1, Time 1; T2, Time 2; SD, Standard deviation; Sig., Statistical significance; DASS21, Depression Anxiety Stress Scales; RSES, Rosenberg Self-Esteem Scale. In bold: p <.05.

### Experiences of family stigma and adolescent well-being

**Table 3** shows the associations between Experiences of Family Stigma and psychological distress (DASS-21 subscales) and self-esteem (RSES) measures among girls, after adjusting for covariates and baseline values of each outcome. The explained variance (adjusted R²) of the multivariate models ranged from 0.27 to 0.34.

**Table 3:** Association between experiences of family stigma (T1–T2) and psychological outcomes at T2 in girls, adjusted for baseline values (T1), age, BMI-z score at T1, European origin, socioeconomic status, and WBISM at T1.

Outcome (T2)	Experiences of family stigma	Adjusted marginal mean	95% CI		Adjusted B	95% CI		Sig.	Adjusted r²
DASS21 Depression								**<.001**	**0.31**
	Never	11.90	10.54	13.27	ref.				
	Only at T1	8.88	5.28	12.48	-3.03	-6.91	0.85	0.126	
	at T1 and T2	14.85	10.62	19.07	2.94	-1.63	7.51	0.206	
	Only at T2	16.47	13.24	19.69	**4.56**	**0.95**	**8.18**	**0.014**	
DASS21 Anxiety								**<.001**	**0.34**
	Never	12.15	10.90	13.40	ref.				
	Only at T1	9.63	5.62	13.64	-2.52	-6.78	1.74	0.245	
	at T1 and T2	14.58	11.33	17.84	2.43	-1.21	6.07	0.190	
	Only at T2	15.85	12.55	19.15	**3.70**	**0.11**	**7.29**	**0.043**	
DASS21 Stress								**<.001**	**0.27**
	Never	14.20	12.91	15.47	ref.				
	Only at T1	13.20	9.35	17.05	-1.00	-5.13	3.12	0.632	
	at T1 and T2	16.81	13.42	20.20	2.61	-1.11	6.33	0.169	
	Only at T2	17.89	15.28	20.50	**3.69**	**0.68**	**6.70**	**0.016**	
DASS21 Total								**<.001**	**0.34**
	Never	38.32	34.80	41.84	ref.				
	Only at T1	31.29	21.27	41.32	-7.03	-17.78	3.73	0.199	
	at T1 and T2	46.05	36.45	55.65	7.73	-2.85	18.31	0.152	
	Only at T2	50.22	42.05	58.38	**11.89**	**2.71**	**21.08**	**0.011**	
RSES								**<.001**	**0.40**
	Never	29.08	28.32	29.85	ref.				
	Only at T1	31.15	28.48	33.81	2.06	-0.74	4.87	0.149	
	at T1 and T2	29.14	26.62	31.66	0.06	-2.60	2.72	0.967	
	Only at T2	28.26	26.52	30.00	-0.82	-2.73	1.08	0.395	

T1, Time 1; T2, Time 2; CI, Confidence Interval; Sig., Statistical significance; DASS21, Depression Anxiety Stress Scales; RSES, Rosenberg Self-Esteem Scale; Ref., reference category. In bold: p <.05.

Overall, a similar pattern was observed across all DASS-21 subscales. Compared to the group that reported no family stigma (Never group), the adjusted mean scores were lower for those who reported experiences Only at T1. Girls exposed to family stigma (at T1 and T2, and Only at T2) reported higher levels of depressive symptoms, anxiety symptoms, and stress, as well as higher total DASS-21 scale scores. However, the highest adjusted mean scores across all DASS-21 scores were observed in girls who reported family stigma only at T2. In this group, the differences were statistically significant for all outcomes (p<.05), and these were the only significant associations found. Regarding RSES, no significant associations were observed in any exposure group.

**Figure 1:**
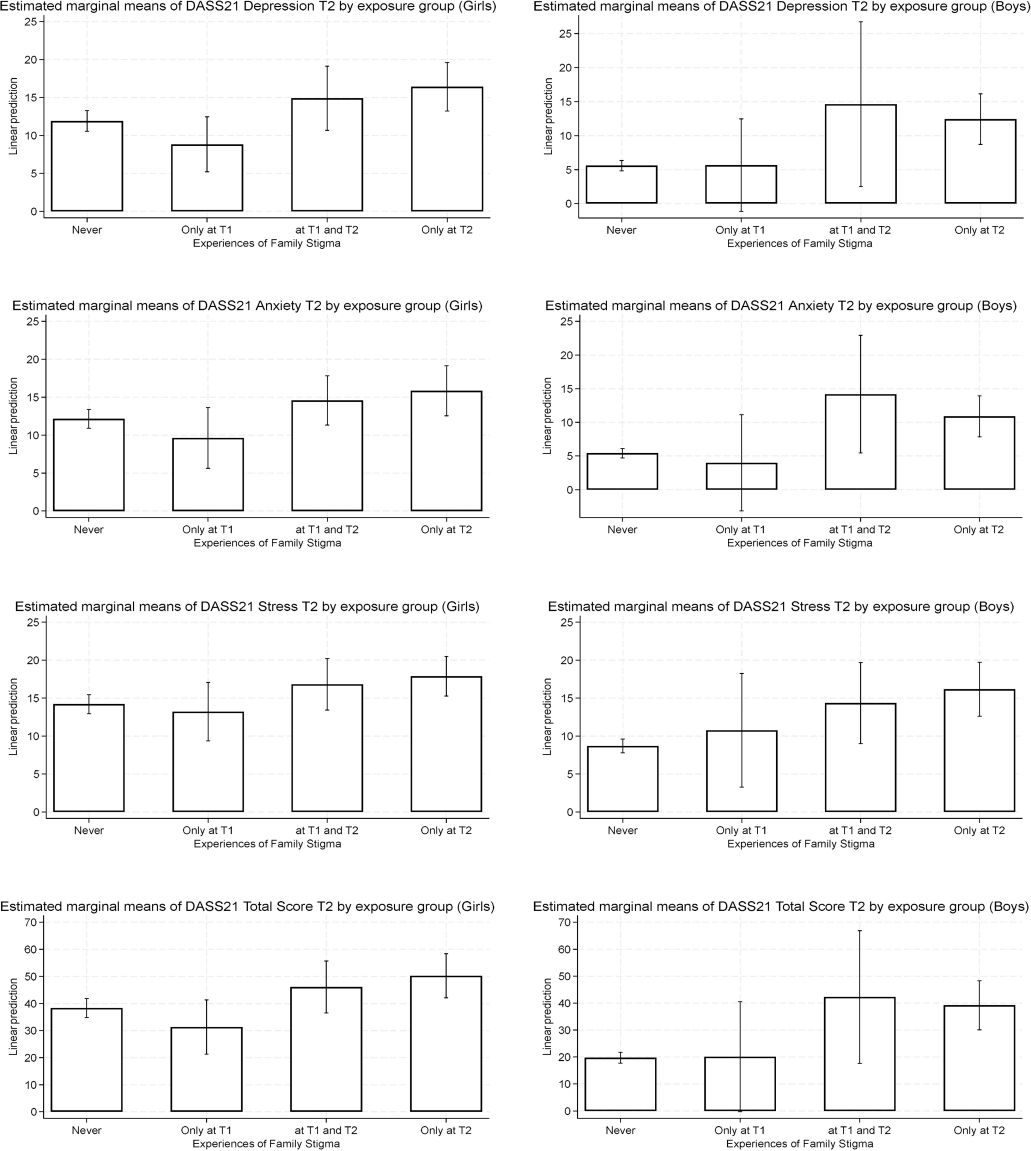
Adjusted estimated means (and 95% confidence intervals) DASS-21 scales by experiences of family stigma exposure.

Given the lower prevalence of reported family stigma across time points among boys, adjusted analyses were not reported for this group. Nevertheless, **Figure 1** includes the estimated marginal means of DASS-21 scales and total scores at T2 by Experiences of Family Stigma exposure for girls and boys, for descriptive and exploratory purposes. Adjusted estimated means suggest a different pattern than those found among girls with smaller differences across exposure categories and lower levels of emotional distress compared to girls. However, these observations should be regarded as exploratory, as the low prevalence of stigma in boys limited statistical power.

### Maternal comments about weight and adolescent well-being

The associations between Maternal Comments about Weight and psychological distress and self-esteem measures among girls, after adjusting for covariates and baseline values of each outcome, are shown in **Table 4**. The explained variance (adjusted R²) of the multivariate models ranged from 0.29 to 0.40. In general, similar trends were observed across the DASS-21 subscales and RSES. Compared to girls who reported no Maternal Comments about Weight (Never group), those who reported such comments at T1 and T2, or Only at T2, showed higher adjusted mean scores on the DASS-21 Depression, Anxiety, Stress, and Total scales. However, statistically significant differences were found only among girls who reported such comments at T2 only (p<.01 to p<.001). Regarding RSES adjusted mean scores, no differences were found among girls who reported Maternal Comments about Weight Only at T1 or at both time points (at T1 and T2), compared to Never group. In contrast, those who reported maternal comments only at T2 showed significantly lower adjusted mean RSES scores.

**Table 4:** Association between maternal comments about weight (T1–T2) and psychological outcomes at T2 in girls, adjusted for baseline values, age, BMI-Z score at T1, European origin, socioeconomic status, and WBISM at T1.

Outcome (T2)	Maternal comments about weight	Adjusted marginal mean	95% CI		B	95% CI		Sig.	Adjusted r²
DASS21 Depression								**<.001**	**0.31**
	Never	11.50	10.01	12.99	Ref.				
	Only at T1	11.67	8.67	14.67	0.17	-3.30	3.64	0.922	
	at T1 and T2	14.62	10.78	18.47	3.12	-1.15	7.40	0.151	
	Only at T2	16.48	13.53	19.43	**4.98**	**1.58**	**8.38**	**0.004**	
DASS21 Anxiety								**<.001**	**0.34**
	Never	11.94	10.61	13.28	Ref.				
	Only at T1	11.18	7.80	14.56	-0.76	-4.47	2.95	0.687	
	at T1 and T2	14.19	10.72	17.65	2.24	-1.65	6.14	0.258	
	Only at T2	16.04	13.35	18.73	**4.10**	**1.07**	**7.13**	**0.008**	
DASS21 Stress								**<.001**	**0.29**
	Never	13.79	12.47	15.11	Ref.				
	Only at T1	14.33	11.34	17.32	0.54	-2.77	3.86	0.747	
	at T1 and T2	16.61	13.63	19.57	2.81	-0.60	6.22	0.105	
	Only at T2	18.68	15.94	21.41	**4.89**	**1.77**	**8.01**	**0.002**	
DASS21 Total								**<.001**	**0.34**
	Never	37.28	33.51	41.04	Ref.				
	Only at T1	37.04	28.64	45.45	-0.23	-9.65	9.18	0.961	
	at T1 and T2	45.37	36.23	55.50	8.09	-2.66	18.44	0.125	
	Only at T2	51.17	43.72	58.50	**13.89**	**5.37**	**22.41**	**0.001**	
RSES								**<.001**	**0.40**
	Never	29.52	28.69	30.35	Ref.				
	Only at T1	29.78	27.77	31.78	0.26	-1.96	2.47	0.820	
	at T1 and T2	28.51	26.31	30.71	-1.01	-3.39	1.36	0.403	
	Only at T2	27.31	25.76	28.87	**-2.21**	**-4.00**	**-0.41**	**0.016**	

T1, Time 1; T2, Time 2; CI, Confidence Interval; Sig., Statistical significance; DASS21, Depression Anxiety Stress Scales; RSES, Rosenberg Self-Esteem Scale; Ref., reference category. In bold: p <.05.

**Table 5** provides the associations between Maternal Comments about Weight and psychological distress and self-esteem outcomes among boys, after adjusting for covariates and baseline values of each outcome. The explained variance (adjusted R²) of the models ranged from 0.24 to 0.36. Nevertheless, this group showed significantly higher adjusted mean scores in the DASS-21 Stress and Total scales compared to those in the Never group (p<.05). No significant associations were found between Maternal Comments about Weight and RSES scores among boys.

**Table 5:** Association between maternal comments about weight (T1–T2) and psychological outcomes at T2 in boys, adjusted for baseline values, age, BMI-Z score at T1, European origin, socioeconomic status and WBISM at T1.

Outcome (T2)	Maternal comments about weight	Adjusted marginal mean	95% CI		B	95% CI		Sig.	Adjusted r²
DASS21 Depression								**<.001**	**0.24**
	Never	5.99	5.14	6.86	Ref.				
	Only at T1	6.91	4.03	9.80	0.92	-2.03	3.87	0.541	
	at T1 and T2	3.91	-2.34	10.17	-2.08	-8.53	4.36	0.525	
	Only at T2	8.51	5.58	11.44	2.51	-0.53	5.55	0.105	
DASS21 Anxiety								**<.001**	**0.26**
	Never	5.94	5.16	6.74	Ref.				
	Only at T1	4.32	1.75	6.89	-1.62	-4.23	0.98	0.221	
	at T1 and T2	3.94	-2.24	10.11	-2.01	-8.36	4.33	0.533	
	Only at T2	8.19	5.73	10.63	2.24	-0.33	4.81	0.087	
DASS21 Stress								**<.001**	**0.27**
	Never	9.00	8.04	9.96	Ref.				
	Only at T1	9.75	6.65	12.85	0.75	-2.50	3.98	0.651	
	at T1 and T2	8.85	2.72	14.97	-0.16	-6.4	6.13	0.961	
	Only at T2	12.85	9.31	16.40	**3.85**	**0.16**	**7.54**	**0.041**	
DASS21 Total								**<.001**	**0.30**
	Never	21.01	18.76	23.25	Ref.				
	Only at T1	20.83	13.37	28.29	-0.18	-7.82	7.46	0.964	
	at T1 and T2	15.39	-2.02	32.80	-5.62	-23.50	12.26	0.537	
	Only at T2	29.63	21.71	37.55	**8.62**	**0.38**	**16.86**	**0.040**	
RSES								**<.001**	**0.33**
	Never	34.08	33.52	34.64	Ref.				
	Only at T1	32.78	31.18	34.38	-1.30	-2.97	0.38	0.128	
	at T1 and T2	35.32	31.75	38.89	1.24	-2.44	4.92	0.508	
	Only at T2	32.74	31.07	34.41	-1.34	-3.11	0.44	0.139	

T1, Time 1; T2, Time 2; CI, Confidence Interval; Sig., Statistical significance; DASS21, Depression Anxiety Stress Scales; RSES, Rosenberg Self-Esteem Scale; Ref., reference category. In bold: p <.05.

**Figure 2:**
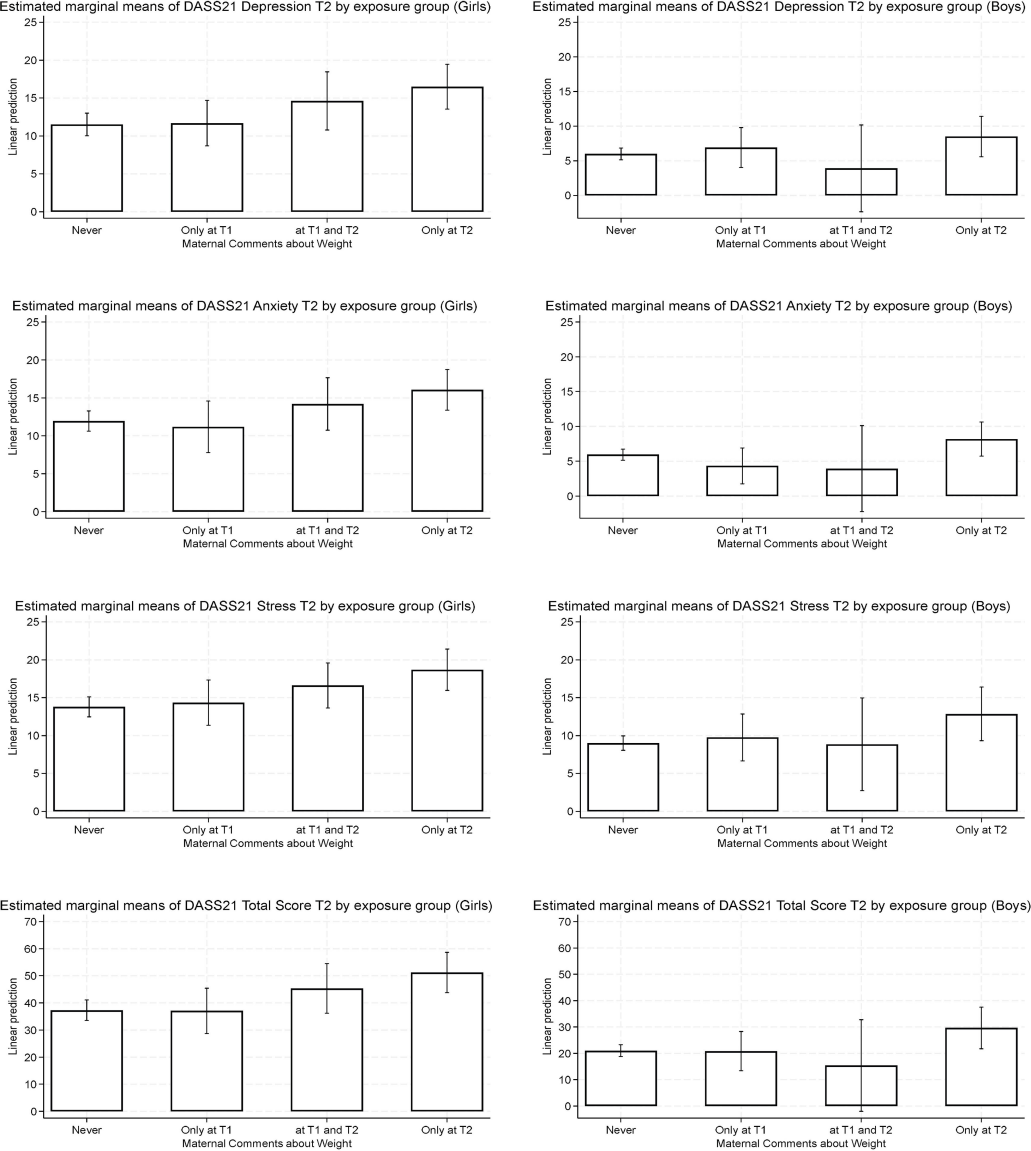
Adjusted estimated means (and 95% confidence intervals) for DASS-21 scales by maternal comments about weight exposure.

As a graphical summary, **Figure 2** presents the estimated marginal means of DASS-21 subscales and total scores at T2 by Maternal Comments about Weight exposure group and gender. Among girls, a consistent pattern is observed whereby those exposed to maternal comments only at T2 showed the highest adjusted mean scores across all outcomes, followed by those exposed at T1 and T2. In contrast, the lowest scores were observed in the Never group. Among boys, no clear or consistent gradient emerged across exposure groups. Across all exposure categories, girls consistently showed higher adjusted mean scores on the DASS-21 subscales compared to boys.

### Paternal comments about weight and adolescent well-being

**Table 6** shows the associations between Paternal Comments about Weight and psychological distress and self-esteem among girls, adjusted for covariates and baseline values of each outcome. The explained variance (adjusted R²) of the multivariate models ranged from 0.28 to 0.40. A general pattern was observed across all adjusted means for the DASS-21 subscales. The lowest adjusted means were found among girls who reported no Paternal Comments about Weight, whereas those who reported such comments at any time point showed higher adjusted mean scores for the DASS-21 subscales, particularly among those exposed only at T2. Compared to the Never group, girls who reported comments only at T2 showed significantly higher adjusted mean scores on the DASS-21 Depression and Total scales (p <.05). No other statistically significant associations were found for girls exposed to paternal comments Only at T1 or at T1 and T2 except fort girls who reported comments Only at T1, who had significantly lower adjusted mean scores on the Stress subscale compared to those in the Never group (p = .020). Regarding RSES, no significant differences in RSES adjusted mean scores were found between groups.

**Table 6:** Association between paternal comments about weight (T1–T2) and psychological outcomes at T2 in girls, adjusted for baseline values, age, BMI-Z score at T1, European origin, socioeconomic status, and WBISM at T1.

Outcome (T2)	Paternal Comments about Weight	Adjusted marginal mean	95% CI		B	95% CI		Sig.	Adjusted R²
**DASS21 Depression**								**<.001**	**0.31**
	Never	12.09	10.84	13.34	Ref.				
	Only at T1	9.82	6.17	13.46	-2.28	-6.20	1.61	0.253	
	at T1 and T2	16.54	10.98	22.10	4.44	-1.29	10.18	0.128	
	Only at T2	17.03	13.04	21.03	**4.94**	**0.70**	**9.18**	**0.023**	
								
**DASS21 Anxiety**								**<.001**	**0.33**
	Never	12.24	11.13	13.34	Ref.				
	Only at T1	11.34	7.70	14.70	-0.90	-4.49	2.69	0.623	
	at T1 and T2	15.10	9.69	20.50	2.86	-2.67	8.38	0.309	
	Only at T2	16.61	12.29	20.92	4.37	-0.10	8.84	0.055	
								
**DASS21 Stress**								**<.001**	**0.28**
	Never	14.75	13.59	15.90	Ref.				
	Only at T1	11.13	8.33	13.92	**-3.62**	**-6.68**	**-0.56**	**0.020**	
	at T1 and T2	17.03	13.04	21.53	2.54	-1.91	6.98	0.262	
	Only at T2	18.22	14.71	21.72	3.47	-0.24	7.18	0.067	
**DASS21 Total**								**<.001**	**0.34**
	Never	39.12	36.00	42.23	Ref.				
	Only at T1	32.07	23.23	40.89	-7.05	-16.54	2.43	0.144	
	at T1 and T2	49.03	34.71	63.36	9.91	-5.80	24.62	0.186	
	Only at T2	51.63	40.66	62.60	**12.51**	**1.03**	**23.99**	**0.033**	
**RSES**								**<.001**	**0.40**
	Never	29.39	28.65	30.13	Ref.				
	Only at T1	29.91	27.85	31.97	0.52	-1.68	2.73	0.641	
	at T1 and T2	27.36	24.24	30.50	-2.02	-5.25	1.20	0.218	
	Only at T2	27.15	24.98	29.32	-2.24	-4.56	0.08	0.058	

T1, Time 1; T2, Time 2; CI, Confidence Interval; Sig., Statistical significance; DASS21, Depression Anxiety Stress Scales; RSES, Rosenberg Self-Esteem Scale; Ref., reference category. In bold: p <.05.

**Table 7** presents the associations between Paternal Comments about Weight and psychological distress and self-esteem among boys, after adjusting for covariates and baseline values of each outcome. The explained variance (adjusted R²) of the models ranged from 0.26 to 0.32. Compared to boys who reported no Paternal Comments about Weight, no significant differences were observed for boys exposed Only at T1 or at T1 and T2 for any of the DASS-21 subscales. However, those who reported paternal comments only at T2 showed significantly higher adjusted mean scores on the DASS-21 Depression, Anxiety, Stress, and Total scales compared to Never group (p<.05). Regarding RSES, no significant differences in adjusted mean scores were found between boys exposed to Paternal Comments about Weight and those who were not exposed.

**Table 7:** Association between paternal comments about weight (T1–T2) and psychological outcomes at T2 in boys, adjusted for baseline values, age, BMI-Z score at T1, European origin, socioeconomic status and WBISM at T1.

Outcome (T2)	Paternal comments about weight	Adjusted marginal mean	95% CI		B	95% CI		Sig.	Adjusted r²
DASS21 Depression								**<.001**	**0.26**
	Never	6.08	5.22	6.94	Ref.				
	Only at T1	4.82	2.74	6.90	-1.26	-3.54	1.02	0.279	
	at T1 and T2	6.90	-0.27	14.07	0.82	-6.45	8.09	0.827	
	Only at T2	11.25	6.55	15.95	**5.17**	**0.38**	**9.96**	**0.034**	
DASS21 Anxiety								**<.001**	**0.29**
	Never	5.89	5.10	6.69	Ref.				
	Only at T1	3.86	1.90	5.83	-2.02	-4.19	0.14	0.067	
	at T1 and T2	4.61	-2.13	11.35	-1.28	-8.11	5.54	0.711	
	Only at T2	10.87	6.92	14.81	**4.98**	**0.89**	**9.06**	**0.017**	
DASS21 Stress								**<.001**	**0.28**
	Never	9.22	8.27	10.18	Ref.				
	Only at T1	8.25	5.63	10.87	-0.97	-3.77	1.82	0.495	
	at T1 and T2	7.79	0.78	14.80	-1.43	-8.56	5.70	0.693	
	Only at T2	15.38	10.43	20.33	**6.16**	**1.05**	**11.26**	**0.018**	
Total DASS21								**<.001**	**0.32**
	Never	21.22	18.99	23.47	Ref.				
	Only at T1	16.88	11.26	22.51	-4.34	-10.46	1.77	0.163	
	at T1 and T2	18.88	-1.40	39.16	-2.35	-22.87	18.17	0.822	
	Only at T2	37.28	25.29	49.27	**16.05**	**3.70**	**28.39**	**0.011**	
RSES								**<.001**	**0.32**
	Never	33.95	33.39	34.52	Ref.				
	Only at T1	33.60	32.24	34.96	-0.35	-1.86	1.16	0.649	
	at T1 and T2	34.07	32.22	35.92	0.12	-1.85	2.09	0.905	
	Only at T2	32.91	30.13	35.70	-1.04	-3.89	1.81	0.475	

T1, Time 1; T2, Time 2; CI, Confidence Interval; Sig., Statistical significance; DASS21, Depression Anxiety Stress Scales; RSES, Rosenberg Self-Esteem Scale; Ref., reference category. In bold: p <.05.

**Figure 3:**
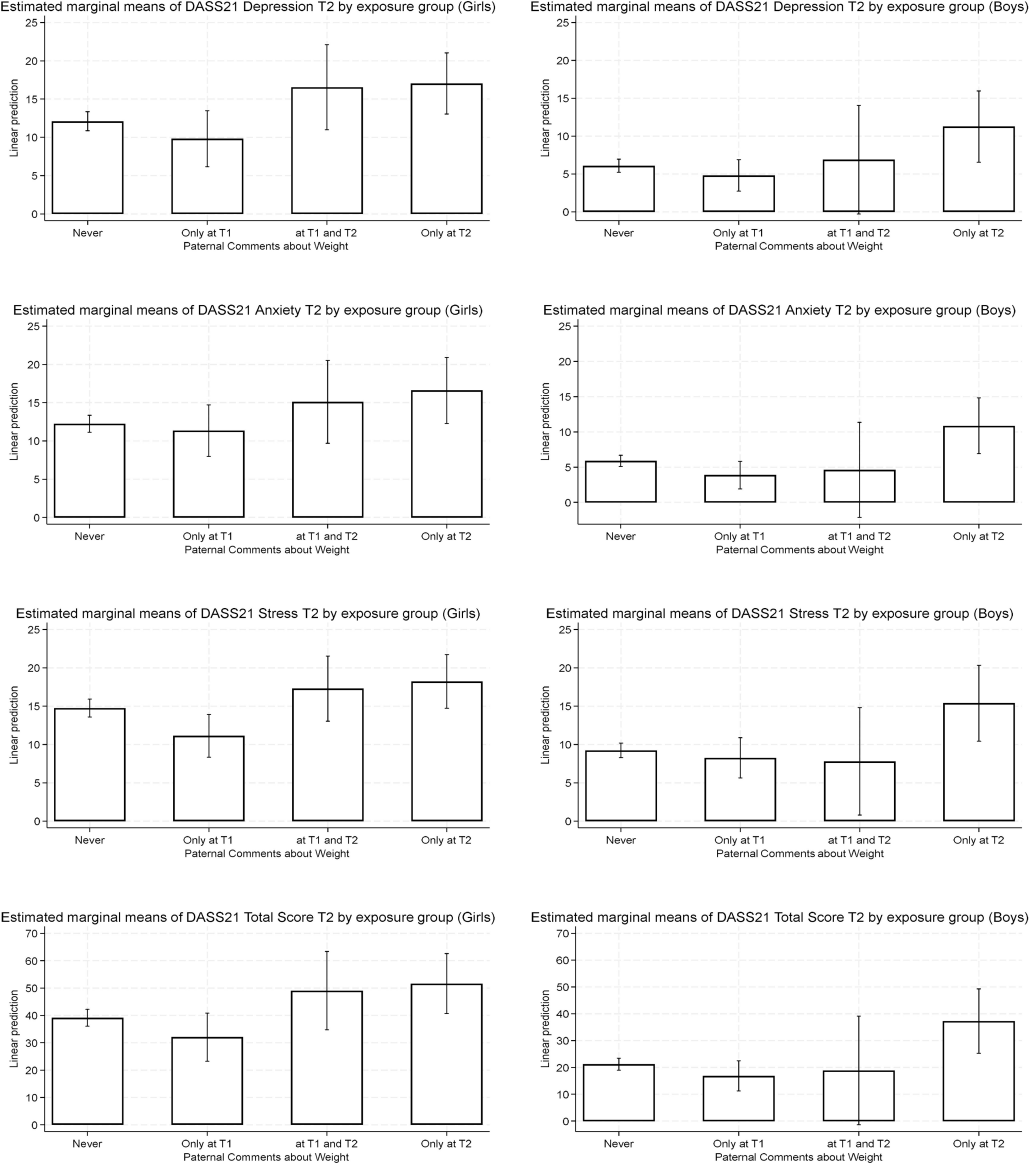
Adjusted estimated means for DASS-21 scales (and 95% confidence intervals) by paternal comments about weight exposure.

**Figure 3** presents a graphical summary of the estimated marginal means of DASS-21 subscales and total scores at T2 by parental comments exposure group and gender. Different gender-specific patterns emerged. Among girls, adjusted mean scores were higher across exposure groups compared to the Never group, with the highest values in those exposed only at T2, while girls exposed Only at T1 had significantly lower Stress scores. Conversely, among boys, lower adjusted mean scores were observed at T1 and T2, and only those exposed Only at T2 showed the highest scores across all DASS-21 outcomes. Overall, girls tended to show higher adjusted scores than boys across outcomes and exposure groups.

### Parental comments about dieting and adolescent well-being

The associations between Parental Comments about Dieting (T1–T2) and psychological outcomes among girls, adjusted for covariates and baseline values of each outcome are presented in **Table 8**. The explained variance (adjusted R²) of the multivariate models ranged from 0.26 to 0.39. Higher adjusted mean scores on the DASS-21 subscales were observed among girls who reported Parental Comments about Dieting at T2 only. For example, compared to the Never group, these girls had higher scores on the DASS-21 Anxiety subscale, although the difference did not reach statistical significance (p = .073). No significant associations were found for girls exposed Only at T1 or both at T1 and T2 for any of the rest of DASS-21 subscales. Regarding self-esteem (RSES), no significant differences in adjusted mean scores were observed between groups.

**Table 8:** Association between parental comments about dieting (T1–T2) and psychological outcomes at T2 in girls, adjusted for baseline values, age, BMI-Z score at T1, European origin, socioeconomic status, and WBISM at T1.

Outcome (T2)	Parental comments about dieting	Adjusted marginal mean	95% CI		B	95% CI		Sig.	Adjusted r²
DASS21 Depression								**<.001**	**0.30**
	Never	12.51	11.17	13.85	Ref.				
	Only at T1	11.59	8.94	14.25	-0.92	-4.01	2.18	0.561	
	at T1 and T2	9.30	3.93	14.68	-3.21	-8.87	2.46	0.266	
	Only at T2	16.20	12.02	20.38	3.69	-0.72	8.11	0.101	
DASS21 Anxiety								**<.001**	**0.33**
	Never	12.52	11.29	13.76	Ref.				
	Only at T1	11.33	8.26	14.41	-1.19	-4.58	2.20	0.490	
	at T1 and T2	12.26	7.11	17.41	-0.26	-5.71	5.18	0.924	
	Only at T2	15.84	12.47	19.21	3.32	-0.31	6.95	0.073	
DASS21 Stress								**<.001**	**0.26**
	Never	14.89	13.64	16.14	Ref.				
	Only at T1	13.64	11.23	16.06	-1.24	-4.01	1.53	0.278	
	at T1 and T2	14.07	8.79	19.36	-0.82	-6.33	4.70	0.597	
	Only at T2	16.55	13.85	19.25	1.67	-1.35	4.68	0.335	
DASS21 Total								**<.001**	**0.33**
	Never	39.97	36.54	43.39	Ref.				
	Only at T1	36.34	29.05	43.61	-3.63	-11.92	4.66	0.389	
	at T1 and T2	35.42	20.60	50.25	-4.55	-20.09	11.00	0.565	
	Only at T2	48.65	39.12	58.17	8.68	-1.55	18.91	0.096	
RSES								**<.001**	**0.39**
	Never	29.31	28.49	30.13	Ref.				
	Only at T1	29.29	27.40	31.17	-0.11	-2.23	2.02	0.921	
	at T1 and T2	29.36	26.97	31.75	0.16	-2.91	2.59	0.909	
	Only at T2	27.37	25.50	29.25	-1.88	-3.98	0.23	0.081	

T1, Time 1; T2, Time 2; CI, Confidence Interval; Sig., Statistical significance; DASS21, Depression Anxiety Stress Scales; RSES, Rosenberg Self-Esteem Scale; Ref., reference category. In bold: p <.05.

**Table 9** presents the associations between Parental Comments about Dieting (T1–T2) and psychological outcomes at T2 among boys, adjusted for covariates and baseline values of each outcome. The explained variance (adjusted R²) of the models ranged from 0.24 to 0.32. Although no significant associations emerged when exposed groups to Parental Comments about Dieting were compared with Never group for any of the DASS-21 subscales, boys who reported parental comments Only at T1 showed lower adjusted mean scores on all DASS-21 subscales, with the difference reaching marginal statistical significance for Depression (p = .060) and Total score (p = .061), while boys who reported parental comments only at T2 tended to have higher mean scores on Stress. Regarding RSES, no significant differences in adjusted mean scores were found between groups.

**Table 9:** Association between parental comments about dieting (T1–T2) and psychological outcomes at T2 in boys, adjusted for baseline values, age, BMI-Z score at T1, European origin, socioeconomic status and WBISM at T1. n=266.

Outcome (T2)	Parental comments about dieting	Adjusted marginal mean	95% CI		B	95% CI		Sig.	Adjusted r²
DASS21 Depression								**<.001**	**0.24**
	Never	6.34	5.37	7.31	Ref.				
	Only at T1	4.03	1.88	6.18	-2.31	-4.71	0.10	0.060	
	at T1 and T2	7.52	3.99	11.04	1.18	-2.55	4.90	0.535	
	Only at T2	7.63	4.81	10.45	1.29	-1.76	4.34	0.405	
DASS21 Anxiety								**<.001**	**0.27**
	Never	5.75	4.84	6.66	Ref.				
	Only at T1	4.25	2.64	5.87	-1.50	-3.39	0.40	0.121	
	at T1 and T2	7.94	4.83	11.05	2.19	-1.09	5.48	0.189	
	Only at T2	8.04	5.39	10.69	2.30	-0.59	5.18	0.119	
DASS21 Stress								**<.001**	**0.27**
	Never	9.30	8.23	10.36	Ref.				
	Only at T1	7.52	4.95	10.09	-1.78	-4.59	1.02	0.212	
	at T1 and T2	9.93	6.66	13.21	0.63	-2.98	4.25	0.729	
	Only at T2	12.89	9.03	16.75	3.59	-0.46	7.64	0.082	
DASS21 Total								**<.001**	**0.30**
	Never	21.40	18.87	23.98	Ref.				
	Only at T1	15.66	10.32	20.93	-5.74	-11.74	0.27	0.061	
	at T1 and T2	25.56	16.58	36.72	4.16	-5.39	13.71	0.392	
	Only at T2	28.53	20.18	36.87	7.12	-1.78	16.02	0.116	
RSES								**<.001**	**0.32**
	Never	34.05	33.46	34.65	Ref.				
	Only at T1	33.33	31.79	34.87	-0.73	-2.37	0.92	0.384	
	at T1 and T2	33.59	31.90	35.28	-0.47	-2.32	1.38	0.620	
	Only at T2	33.17	30.89	35.45	-0.89	-3.32	1.54	0.473	

T1, Time 1; T2, Time 2; CI, Confidence Interval; Sig., Statistical significance; DASS21, Depression Anxiety Stress Scales; RSES, Rosenberg Self-Esteem Scale, Ref., reference category. In bold: p <.05.

## Discussion

This longitudinal study examined how adolescents’ reports of family-based weight stigma is associated with their psychological well-being two years later, with particular attention to differences based on parent type and adolescent gender. Additionally, the study provided insights into how both the recency and cumulative exposure to weight stigma within the family context affect mental health outcomes over time. These findings support prior calls for further research into the temporal dynamics of parental weight-related comments and their impact on adolescent well-being, i.e., whether adult psychological effects stem from early-life exposure, more recent experiences, or the cumulative burden of recurrent stigma (23), given that, to our knowledge, no prior research explicitly differentiates recent from cumulative family-based weight stigma exposure. Overall, our framing acknowledges robust dose–response links between adversity and health, uses sensitization and desensitization as exploratory lenses, and emphasizes that the developmental timing of exposure (i.e., *when* during adolescence stigma occurs) may be as consequential as its cumulative burden (i.e., *how much* stigma is experienced).

Before interpreting specific influences, we first situate the sample’s DASS-21 and RSES scores using Spanish reference values. Benchmarking our adjusted marginal means against Spanish DASS-21 patient norms (49), scores were generally below the patient mean across Depression, Anxiety, and Stress, with one exception: Anxiety among girls exposed to familial weight stigma or parental weight-related comments at T2 approached or slightly exceeded the patient mean. These comparisons are indicative rather than diagnostic because validated Spanish cut-offs or minimal clinically important differences (MCIDs) for DASS-21 are unavailable; moreover, youth internalizing symptoms have risen in recent years —particularly post-COVID-19 and among adolescent girls— so contemporary baselines may exceed 2005 norms (54). Regarding self-esteem, the Spanish validation of the RSES (51) (Martín-Albo et al., 2017) reports only sex-specific means (men: M = 32.53, SD = 3.92; women: M = 31.14, SD = 4.51) and no clinical cut-offs, precluding clinical interpretation of RSES scores in our sample.

### Longitudinal associations between family-based weight stigma and adolescent well-being

The proportion of adolescents reporting family-based weight stigma (6-13% among girls and 1-7% among boys depending on the time point assessed) was relatively low compared to prior literature (23, 55) and cumulative exposure across both waves was uncommon. As regards the longitudinal association between experiences of family stigma and adolescents’ psychological distress, the finding that the highest levels of distress were observed among girls exposed only at T2 suggests that recent exposure may have a stronger emotional impact than earlier or cumulative experiences, underscoring the acute influence of current family dynamics during mid-adolescence. These temporal patterns are further discussed in a later section.

### Longitudinal associations between parental comments about weight and adolescent well-being

Findings indicate that mothers were more frequently identified as sources of stigmatizing weight-related comments than fathers, at either T1 or T2, consistent with previous research suggesting that, within families, such comments tend to be more prevalent from mothers than from fathers (23, 55). In examining the associations between family-based weight stigma and adolescents’ psychological well-being, the strongest associations for girls were observed when exposure occurred only at T2, with higher levels of stress, anxiety, depression, and lower self-esteem, compared to those who never reported maternal stigma. By contrast, earlier (Only at T1) or cumulative (at T1 and T2) exposure did not differ significantly from the Never group. These patterns suggest that recent exposure may have a greater impact than earlier or cumulative exposure. Among boys, maternal stigma was specifically associated with elevated stress, both on the DASS Stress subscale and Total score, with stronger effects observed for those recently exposed (Only at T2) compared to the never exposed. Similarly, among girls, paternal weight-related stigma was linked to higher depression –both on the DASS depression subscale and total score–, relative to the Never group. For boys, paternal stigma followed a comparable pattern, with recent exposure associated with higher scores across all DASS subscales compared to the never exposed. Our findings are consistent with prior longitudinal research demonstrating that family-based weight stigma is associated with a deterioration in psychological well-being over time, including higher stress, depressive symptoms, and WBI, as well as lower self-esteem and body appreciation in adolescent and emerging adult populations (18, 27, 56).

The different models accounted for a moderate proportion of the variance in psychological outcomes, with greater explanatory power observed among girls, particularly in models involving maternal comments and paternal comments. Additional covariates, such as baseline levels of WBI and zBMI, also made significant contributions to the prediction of anxiety, depression, stress, and self-esteem, particularly among female participants. Regarding WBI, extensive literature supports the notion that experiences of weight stigma are exacerbated when internalized, leading to greater psychological distress (13), and that WBI may mediate the relationship between weight stigma and psychological outcomes (57). Additionally, according to previous studies, more frequent negative weight-related comments from parents were associated with higher levels of WBI, regardless of whether they came from mothers or fathers. Conversely, positive comments were linked to lower levels of WBI and greater body appreciation among adolescents (17). Weight status may function both as a risk factor for exposure to family-based stigma (25) and as an independent predictor of emotional difficulties (58), possibly due to the increased salience of body weight in the context of thin ideal internalization, peer interactions, and adolescents’ self-concept (59). Accordingly, in our study, weight status appeared to play a more direct and pervasive role in adolescent psychological well-being than parental weight-related comments alone.

### Longitudinal associations between parental encouragement to diet and adolescent well-being

Regarding parental encouragement to diet, no significant effects of this variable were found on any of the dependent variables in either boys or girls. This finding contrasts with the majority of previous studies, which have shown that parental encouragement to diet predicts children’s dieting behaviors (20), with some also suggesting a gender-linked transmission pattern in which mothers are more likely to influence daughters (25, 55). It is important to note, however, that the present study focused on psychological well-being rather than weight-control behaviors. It is possible that the psychological impact of such encouragement may take longer to emerge, or that it may be attenuated by competing sociocultural influences during adolescence, such as peer dynamics and social media exposure (60, 61). This null effect could also be related to the limited sensitivity of the measurement tool used (single-item with binary response).

### Recency and cumulative exposure to family-based weight stigma

In terms of temporal dynamics, the evidence from this study suggests that recent exposure to family-based weight stigma may have a more immediate and pronounced effect on adolescents’ psychological well-being than earlier or cumulative exposure. This pattern stands in contrast to previous research highlighting the cumulative impact of weight-related pressures —such as parental encouragement to diet— on long-term health outcomes. For instance, in a study by Berge and colleagues (29), each occurrence of encouragement to diet from close relationships between Time 1 and 3 was associated with a 17% increased risk of binge eating at Time 4 in females and a 39% increase in males, even after adjusting for baseline BMI, underscoring the lasting influence of repeated weight-related messaging on disordered eating behaviors. Moreover, the persistence of weight stigma experiences across adolescence and young adulthood is well established. Eisenberg and colleagues (62) found that family-based weight stigma can persist over time: adolescents who experienced weight-related teasing from family members were twice as likely to report similar hurtful comments ten years later, independent of gender, race, socioeconomic status, or weight change. Haines et al. (38) similarly found that the prevalence of weight-related teasing remained high and relatively stable from adolescence into young adulthood, with one exception: males first assessed in early adolescence, among whom teasing rates increased over time. Although neither study directly assessed the psychological impact of persistent weight-related teasing, the observed stability in stigma exposure may reflect desensitization/habituation processes, whereby repeated exposure leads to a decreased emotional response over time while, in line with the sensitization theory, more recent or novel experiences of stigma may elicit a stronger psychological impact. In our study, the lack of significant associations for cumulative exposure (at T1 and T2) suggests that the timing of exposure may play a more critical role than its duration. Specifically, recent exposure appeared to be associated with a stronger emotional response than chronic or earlier experiences, raising the possibility that repeated stigma may lead to psychological adaptation, reducing its perceived salience over time. This hypothesis warrants further empirical exploration.

When interpreting the findings related to the heightened psychological impact of recent exposure at T2, it is important to consider participants’ developmental stage. At T1, participants were in early-to-mid adolescence. However, at T2, the majority of participants were in mid-to-late adolescence (mean age was approximately 16 years), a developmental period characterized by heightened sensitivity to social evaluation and body image concerns, as well as a critical window for identity formation. Exposure to family-based weight stigma during this stage may therefore interfere with the development of a positive body image and a stable self-concept, ultimately contributing to a decline in psychological well-being (63). However, these findings contrast with those of a recent meta-analysis (58), which concluded that younger age moderated the association between weight stigma and mental health, with stronger effects observed in younger populations. One explanation proposed by Warnick and colleagues (9) is that younger children may be especially vulnerable to the psychological effects of weight stigma due to their limited coping skills and lack of prior experience in managing social stressors such as peer or family-based victimization. In any case, the relationship between experiences of weight stigma and health outcomes across developmental stages remains underexplored (35). Future research should further examine how the timing of parental weight-related comments shapes adolescent well-being.

### Gender differences

Our findings align with previous research, showing that girls reported higher overall exposure to family-based weight stigma —especially at T2— and exhibited greater psychological vulnerability compared to boys. This may be linked to girls’ heightened sensitivity to, and greater likelihood of reporting weight-related comments from family members -particularly from mothers- which have been more strongly associated with depressive symptoms in this group (17, 62). Puhl and colleagues (25) further note that such stigma often occurs more frequently within same-gender parent–child dyads. It should be noted that the very low prevalence of reported family stigma among boys substantially limited the statistical power of the analyses. As a result, gender comparisons and the observed trends in boys should be considered exploratory only. In our study, maternal comments about weight, both recent and cumulative, were more frequently reported by girls than by boys, and recent maternal comments (T2) emerged as the strongest predictor of psychological distress among adolescent girls. This aligns with prior evidence showing that mothers are more likely than fathers to engage in weight-focused conversations with their children (18, 23). However, emerging literature highlights that when fathers do participate in such conversations, their comments may have particularly strong associations with adverse outcomes in emerging adults (17, 18). Despite the historical underrepresentation of fathers in research on parent–adolescent weight communication (64), available evidence underscores that both maternal and paternal input can have meaningful impacts, though they may differ in frequency, content, and psychological consequences.

It is important to acknowledge both the strengths and limitations of this study. Strengths include a large, population-based adolescent cohort from a Mediterranean context, where evidence on family-based weight stigma remains limited, enhancing generalizability to similar settings. The prospective, two-wave design allowed us to implement a time-sensitive framework that explicitly contrasts recent (Only at T2) versus cumulative (at T1 and T2) exposure using a transparent four-category scheme. We conducted gender-sensitive analyses by examining maternal and paternal comments separately and reporting results for girls and boys, and we adjusted for key covariates (age, origin, socioeconomic status, zBMI, baseline WBI) to reduce the influence of potential confounding variables. Finally, by objectively measuring the weight and height of participants, the accuracy of weight status estimation is ensured, whereas most community studies on this topic rely on self-reported data.

However, several limitations should be considered when interpreting these findings. First, all data were obtained through adolescent self-report, which may be subject to social desirability bias and potential perceptual or recall biases, particularly for sensitive topics like family-based weight stigma. Second, the assessment of parental weight-related comments and encouragement to diet relied on a limited number of binary-coded items, which can undermine measurement reliability and construct validity. Additionally, they did not fully capture the complexity of family communication around weight. Future work should focus on developing validated multi-item scales for assessing this variable. Moreover, this study focused on negative parental communication and did not adequately consider the potential protective effects of positive weight-related messages –shown to be impactful in previous research (25)– or the role of adolescents’ coping strategies in response to family-based weight stigma. Both factors may have buffered the psychological impact in our sample and thus warrant further investigation. In addition, because validated Spanish DASS-21 cut-offs/MCIDs are unavailable, we cannot estimate the proportion of adolescents with clinically meaningful symptoms; thus, clinical inferences should be interpreted with caution. Finally, as with many longitudinal studies, there was a notable loss of participants between the two assessment points; however, among adolescents who remained in the Spanish mandatory secondary education system –the study´s primary target population–, the retention rate exceeded 80%. In addition, sample sizes for several exposure categories were low even among girls, and particularly low among boys. This limited prevalence reduced statistical power across models and precluded testing formal interaction effects with gender.

Future research should address these limitations by developing and using more comprehensive, culturally validated instruments in Spanish to assess the full spectrum of parental communication about weight. It is crucial to include both negative and positive parental messages –for example, encouragement to eat more healthily, engage in physical activity, or participate in family meals– to better understand the unique and longitudinal effect of each variable on adolescent well-being. Equally important is examining how adolescents perceive these messages, as the same comment may be interpreted as supportive by some and stigmatizing by others. Although we included WBI as a covariate, future studies should conduct additional analyses to assess its potential mediating role in the observed associations between parental comments about weight and psychological well-being. Additionally, future studies should examine adolescents’ differential coping strategies in response to both recent and cumulative experiences of family-based weight stigma. This is particularly important given that our results, which contrast with previous evidence highlighting the negative effects of cumulative weight stigma within families, suggest a possible role for desensitization or psychological adaptation.

## Conclusions

Given the negative impact of recent family-based weight stigma on adolescent well-being observed in this longitudinal study, particularly among girls and when maternal and paternal comments are involved, our findings underscore the importance of implementing preventive strategies before the onset of mid-to-late adolescence, when these effects appear to be most pronounced. These results highlight the need for targeted efforts, particularly within pediatric healthcare and school settings, to educate parents on how to recognize and avoid stigmatizing weight-related comments. Instead, they should be encouraged to promote positive, health-focused messages that prioritize well-being over weight and support the adoption of healthy behaviors within the entire family unit, with parents serving as role models. This approach, which is already supported by national guidelines in Spain (65), may be more feasible than attempting to modify deeply rooted parenting styles. Finally, our findings emphasize the importance of considering gender-specific dynamics such as the differing roles of mothers and fathers, and the gender of the adolescent, in the context of family-based weight stigma. Tailoring interventions to account for these nuanced influences may enhance their effectiveness.

## Data Availability

The raw data supporting the conclusions of this article will be made available by the authors, without undue reservation.
